# Physiological dynamics, reproduction‐maintenance allocations, and life history evolution

**DOI:** 10.1002/ece3.5477

**Published:** 2019-07-30

**Authors:** Sinead English, Michael B. Bonsall

**Affiliations:** ^1^ School of Biological Sciences University of Bristol Bristol UK; ^2^ Department of Zoology University of Oxford Oxford UK

**Keywords:** Euler–Lotka, force of selection, levels of fitness, reproductive value, theoretical evolutionary ecology

## Abstract

Allocation of resources to competing processes of growth, maintenance, or reproduction is arguably a key process driving the physiology of life history trade‐offs and has been shown to affect immune defenses, the evolution of aging, and the evolutionary ecology of offspring quality. Here, we develop a framework to investigate the evolutionary consequences of physiological dynamics by developing theory linking reproductive cell dynamics and components of fitness associated with costly resource allocation decisions to broader life history consequences. We scale these reproductive cell allocation decisions to population‐level survival and fecundity using a life history approach and explore the effects of investment in reproduction or tissue‐specific repair (somatic or reproductive) on the force of selection, reproductive effort, and resource allocation decisions. At the cellular level, we show that investment in protecting reproductive cells increases fitness when reproductive cell maturation rate is high or reproductive cell death is high. At the population level, life history fitness measures show that cellular protection increases reproductive value by differential investment in somatic or reproductive cells and the optimal allocation of resources to reproduction is moulded by this level of investment. Our model provides a framework to understand the evolutionary consequences of physiological processes underlying trade‐offs and highlights the insights to be gained from considering fitness at multiple levels, from cell dynamics through to population growth.

## 1 INTRODUCTION

Population biology has long considered the role of individual variation on the dynamics of strategies and dynamics of populations (Lomnicki, 1988). Linking individual‐level variation to population‐level variation (Auslander, Oster, & Huffaker, 1974; de Roos, 1988; Metz & Diekmann, 1986) has tended to focus predominately on the population ecology rather than the evolutionary outcomes (Metz & Diekmann, 1986). Life history approaches to physiological ecology have focused on the role of trade‐offs (Sibly, 1991), and extending this theme, Calow (2008) provides a more contemporary overview of linking physiological processes to ecological evolutionary outcomes such as optimal energy efficiencies, resource allocation, patterns of growth and biochemical adaptations. Derived from the fundamental demographic processes of birth, death and dispersal, life history theory allows the role of fitness, adaptation and constraints on natural selection to be determined (Charlesworth, 1994; Gadgil & Bossert, 1970; Stearns, 1992). Costs and constraints on fitness arise as investment in different traits is mutually exclusive. This gives rise to trade‐offs (Stearns, 1989). As these trade‐offs manifest through survival and reproduction, life history theory provides a framework for measuring relative fitness, and hence adaptations.

One fundamental trade‐off is that reproduction compromises survival, and with limited resources, organisms are faced with decisions on whether to invest in maintenance, repair, or growth. Resource allocation constraints to reproduction and somatic cell maintenance have been shown to influence life history evolution particularly the evolutionary dynamics of senescence (Cichoń, 1997; Kirkwood, 2005; Mangel, 2008; Schoen & Ashman, 1995). Trade‐offs at the physiological level translate differences in the genotype to differences in components of life histories, and ultimately to differences in overall fitness (Harshman & Zera, 2007; McNamara & Houston, 2009; Zera & Harshman, 2001). Limited resources alter energy allocation to physiologies and influence processes such as energy storage, metabolism, oxidative damage, and immune function. For instance, Cox et al. (2010) showed that by altering resource investment in reproduction (in lizards), the physiological costs (in terms of energy storage, immune function, and levels of disease) influencing the trade‐off between reproduction and survival could be quantified. This link with physiology has important consequences for life histories and trait trade‐offs. At the physiological level, variation in life history trade‐offs is likely regulated through specific nutrient limitations and how these resources are allocated (Cotter, Ward, & Kilner, 2011; Rapkin, Jensen, House, Wilson, & Hunt, 2018). Pleiotropic physiological mechanisms might regulate resource allocation between traits (such as reproduction and immune function) within individuals. Genetic variation in such mechanisms at the population level can have significant evolutionary consequences (Schwenke, Lazzaro, & Wolfner, 2016).

If variation in life history trade‐offs occurs through variation in these sorts of physiological mechanisms, then modeling the relationship between physiology and life history requires a dynamical (rather than a fixed geometrical) framework. Geometric frameworks rely on fixed effects approaches (e.g., variance‐covariance decompositions, response surface methodologies) whereas dynamics involve processes with a critical temporal axis. Physiology changes through time. For example, Sudyka, Casasole, Rutkowska, and Cichoń (2016) argue that the lack of an effect between reproductive costs and oxidative stress in zebra finches suggests that physiological function alters the temporal dynamics of protection against free radicals. Similarly, Lan Smith, Merico, Hohn, and Brandt (2014) show that size‐structuring in phytoplankton communities is driven by dynamical physiological allocation patterns of nutrient uptake and allocation to different enzymatic processes affecting life history traits. The temporal dynamics of resource allocation and physiology are integral to the emergence of life history trait trade‐offs. Dynamic resource allocation models have been described (Perrin & Sibly, 1993; Taylor, Gourley, Lawerence, & Kaplan, 1974) yet have not considered, to our knowledge, an explicit scaling up from cellular energy allocation dynamics to life history patterns.

Here, we develop a theoretical approach to link the dynamics of a physiological process (cellular protection) to its effects on life history evolution through survival and reproduction. As outlined, life history theory provides a logical framework in which to develop predictions on allocation decisions and their evolutionary consequences. By decomposing an organism's life history into distinct stages, we can investigate the consequences of resource allocation decisions to reproductive cell production and/or cell maintenance on survival and fecundity schedules. This allows appropriate life history metrics for ecological and evolutionary success to be derived. In our framework, an organism allocates resources to either somatic or reproductive tissues and we investigate how these allocation decisions scale to influence life history evolution.

This approach to linking dynamics across scales of organization has implications for appropriately assessing fitness: it is important not to conflate a component of fitness with the ultimate success of a life history strategy. Determining fitness at different scales provides details on the processes of selection, and we investigate this here by linking both reproductive cell allocation measures and life history measures of fitness. We begin by defining the reproductive cell dynamics and a component of fitness before exploring the full life history consequences of allocation decisions using the Euler–Lotka equation. In contrast to previous models on the implication of cellular damage accumulation and repair (Lee, Metcalfe, Monaghan, & Mangel, 2011; Yearsley et al., 2005), our approach considers the dynamics of reproductive cell formation and loss, and scales these dynamics to investigate the implications on survival and fecundity schedules. More specifically, we derive theoretical conditions for life history evolution (force of selection, reproductive vale, and life history strategies) and discuss the importance of linking physiological processes to broader evolutionary phenomena.

## 2 MODEL FORMULATION

In this section, we outline the reproductive cell allocation model and the life history framework (Table 1 provides an overview of the variables and parameters used).

**Table 1:** Parameter definitions used in the reproductive cell allocation model (Equations 1 and 2) and Euler–Lotka framework (Equation 5)

Reproductive cell model	Definition
λ	Cellular fitness
$V_{0}$	Precursor cell numbers
$V_{n}$	Functional cell numbers
R	Available resources
q	Proportion of resource allocated to somatic cell production
b	Level of protection of reproductive cells
$\mu_{0}$	Rate of death (apoptosis) of precursor reproductive cells
$\mu_{n}$	Rate of death (apoptosis) of functional reproductive cells
$\gamma_{0}$	Maturation rate

Life history model	Definition
r	Intrinsic rate of increase (lifetime fitness)
a	Level of protection of somatic cells
ψ	Scaling parameter linking *V*_n_ to costs on survival
x	Age
m(x)	Fecundity at age x
l(x)	Survival to age x

### 2.1 Reproductive allocation dynamics

**Figure 1:**
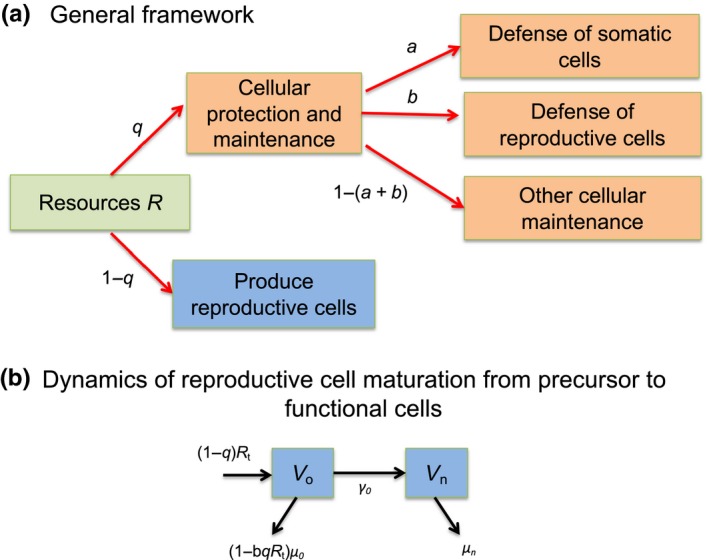
Model formulation. Schematic outline of (A) the general framework for the life history model. Resources are allocated to somatic or reproductive cells, somatic cells produce defenses that protect somatic, or precursor reproductive cells. (B) Dynamics of reproductive cell maturation from precursor to functional cells

To investigate changes in reproductive cells, we consider a limiting resource (R) that can be allocated with proportion q to maintenance and related functions or (1-q) to reproductive cell production (Figure 1A). Of the fraction of resource (q) allocated to cell maintenance, a proportion a is allocated to protecting or repairing somatic cells, b to protecting or repairing reproductive cells, and the remainder (1-a-b) to other cellular functions (which we term “physiological demand”).

In terms of reproductive allocation, we assume that this resource is used in biochemical reactions to convert precursor reproductive cells ($V_{0}$) into functional reproductive cells ($V_{n}$) at a constant rate $\gamma_{0}$ and that cellular protection (at rate qbR) can increase the baseline survival (reduce the mortality $\mu_{0}$) of precursor reproductive cells (Figure 1B).

We describe this biology with the following set of equations:

(1) $$\frac{dV_{0}}{dt}=\left(1-q\right)R_{\left(t\right)}-\gamma_{0}V_{0(t)}-\mu_{0}\left(1-bqR_{\left(t\right)}\right)V_{0(t)}$$

(2) $$\frac{dV_{n}}{dt}=\gamma_{0}V_{0(t)}-\mu_{n}V_{n(t)}$$

where $\mu_{n}$ is the rate of cell death (apoptosis) of functional reproductive cells. (Table 1).

From Equations (1 and 2), the equilibrium state of precursor and functional reproductive cells is as follows:

(3) $$V_{0}=\frac{\left(1-q\right)R}{\gamma_{0}+\mu_{0}\left(1-bqR\right)}$$

(4) $$V_{n}=\frac{\gamma_{0}V_{0}}{\mu_{n}}$$

This equilibrium state is stable when $\mu_{n}+\gamma_{0}+\mu_{0}\left(1-bqR\right)>0$ and $\mu_{n}\left(\mu_{0}\left(1-bqR\right)+\gamma_{0}\right)>0$ (Appendix).

### 2.2 Life history evolution

To quantify the fitness consequences of resource allocation and damage, we use a life history framework. By choosing this approach, we need to define an appropriate measure for fitness. As noted, fitness can be measured at different levels of biological organization and as such many studies only measure a component of fitness and equate this with evolutionary success. The ultimate measure of fitness is a metric that determines whether a strategy will spread or not. Life history theory (developed by Fisher (1930) and Lotka (1939)) provides a basis from which predictive statements on strategy success can be drawn. This theory, centered around the Euler–Lotka equation, defines fitness in terms of the intrinsic rate of increase (r) (Table 1).

The discrete version of the Euler–Lotka equation is as follows:

(5) $$\sum_{x=1}^{\infty }\exp\left(-rx\right)l\left(x\right)m\left(x\right)=1$$

where l(x) is survival to age x and m(x) is the reproduction at age x (Table 1). Age‐specific survival ($s_{j}$) is defined as the product of the probability of surviving from age x to x+1:

(6) $$l\left(x\right)=\prod_{j=1}^{x-1}s_{j}.$$

The age‐specific survival probabilities are determined by the instantaneous death rate between age *x* and *x* + 1:

(7) $$s_{x}=\exp\left(-\int_{\tau }^{\tau +1}\mu \left(x\right)dx\right).$$

Here we define the instantaneous death rate between age classes as costs associated with both the loss of somatic cell function, which is a function of resource allocation at age x, f(R), and the costs of reproduction, a function of the number of precursor reproductive cells, $g(V_{0})$. More explicitly, the age‐specific survival probabilities are determined from:

(8) $$s_{x}=\exp\left(-\int_{\tau }^{\tau +1}f\left(R(x)\right)+g\left(V_{0}\left(x\right)\right)\right)dx$$

(9) $$=\exp\left(-\left(f(R(x)\right)+g\left(V_{0}\left(x\right)\right)\right)$$

where the effects of resources on survival, (f(R(x)), is a diminishing function ($\frac{1}{aqR(x)}$), $g(V_{0})$ is $\psi V_{0}\left(x\right)$, a is the investment in protection of somatic cells (a+b≤1) and ψ is a scaling factor linking precursor reproductive cell numbers to costs on survival.

Reproduction at age *x* is defined as the expected number of functional reproductive cells at age *x* (from Equation 4):

(10) $$V_{n}\left(x\right)=\frac{\gamma_{0}V_{0}\left(x\right)}{\mu_{n}}$$

where $V_{0}\left(x\right)$ is the expected number of precursor cells at age, x given by $V_{0}\left(x\right)=\frac{\left(1-q)R(x\right)}{\gamma_{0}+\mu_{0}\left(1-bqR\left(x\right)\right)}$.

### 2.3 Analysis

Model analysis proceeds by defining and investigating the component of fitness associated with the reproductive cell dynamics (cellular fitness). This only provides a partial understanding of the evolution of cellular protection as it fails to account for survival differences across the life history. To address this and link the cellular dynamics to life histories, we explore the effects of cellular protection on life histories by investigating the force of selection and reproductive value under both increasing (positive rates of increase) and declining (negative rates of increase) population dynamics (Charlesworth, 1994; Fisher, 1930; Keyfitz & Caswell, 2005). We solve the Euler–Lotka expression (Equation 5) for the lifetime fitness measure (r) under different life history parameters associated with providing protection to somatic (a) or reproductive (b) cells, proportion of resource allocated between production of reproductive cells and repair/maintenance of existing cells (q) and the pattern of resource availabilities at age x (e.g., the schedule of declining resources). Specifically, we consider resource declines which are linear, accelerating (exponential), or decelerating (1-exp(-x)).

## 3 RESULTS

### 3.1 Reproductive allocation dynamics

**Figure 2:**
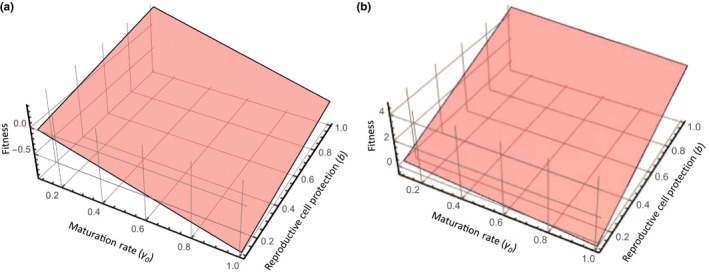
Effects of cell mortality, maturation rate, and defense investment on reproductive cell fitness. Fitness (λ) is expressed in terms of maturation rate ($\gamma_{0}$) and investment in precursor cell protection (b) for different levels of precursor cell death rate ($\mu_{0}$). (A) Under low cell death rates ($\mu_{0}=0.01$) investment in defense has positive fitness benefits when reproductive cell precursor maturation rate is low. (B) When cell death rate is high ($\mu_{0}=0.1$) high investment in protection increases fitness across a range of maturation rates. [Other parameters: R=100, q=0.5]

Reproductive cell fitness (λ) can be determined from Equations (1 and 2) and expressed in terms of a net growth rate (Appendix):

(11) $$\lambda =\left(bqR-1\right)\mu_{0}-\gamma_{0}$$

such that positive fitness occurs when λ>0. Under low cell mortality (Figure 2A), only high levels of investment in protecting reproductive cells (b>0.5) and low maturation rates (long development times) have fitness benefits (λ>0). When cell death rates are high (Figure 2B), greater fitness benefits occur when there is greater investment in reproductive cell protection (b) for a range of maturation rates. This occurs through the multiplicative effects of resource availability and resource allocation (bqR) that offset high levels of intrinsic precursor cell mortality.

In the next sections, we link these findings to investigate how investment in cellular protection affects life histories. We begin by deriving consequences of cellular protection on the force of selection and reproductive value (i.e., *within life histories*). We then extend these results to investigate how lifetime fitness varies *across life histories*, when cellular protection varies.

### 3.2 Within life history optima

#### 3.2.1 Force of selection

In general, the force of selection measures how lifetime fitness changes with respect to a particular trait and this can often be evaluated as a (scaled) ratio between the net reproductive rate and the mean age of reproduction (Charlesworth, 1994; Fisher, 1930). This change in lifetime fitness with respect to changes in the amount of cellular protection to somatic and reproductive cells can be determined using standard methods of differentiation (Appendix).

The force of selection on protection of somatic cells (the level of a) is as follows:

(12) $$\frac{\partial r}{\partial a}=\frac{\sum_{i=1}^{\infty }\exp\left(-rx\right)\exp\left[-\left(\frac{1}{aqR(x)}+\psi V_{0}\left(x\right)\right)\right]V_{n}\left(x\right)}{a^{2}qR\left(x\right)\sum_{i=1}^{\infty }x\exp\left(-rx\right)\exp\left[-\left(\frac{1}{aqR(x)}+\psi V_{0}\left(x\right)\right)\right]V_{n}\left(x\right)}.$$

The numerator is a measure of the net reproductive rate scaled by exp(-rx). The denominator is a measure of the expected age of reproduction again scaled by exp(-rx) and also the level of protection to somatic cells (a), resources allocated to cellular maintenance, (q) and the amount of resource available at age x. A limiting case (Appendix) reveals that this is a declining function in the protection allocated to somatic cells—predicting strong selection for low levels of somatic cell protection and weak selection for high levels of somatic cell protection.

The change in lifetime fitness with respect to changes in the amount of protection provided to precursor reproductive cells (the level of b) is as follows:

(13) $$\frac{\partial r}{\partial b}=-\frac{\sum_{i=1}^{\infty }\exp\left(-rx\right)\exp\left[-\left(\frac{1}{aqR(x)}+\psi V_{0}\left(x\right)\right)\right]\left(\frac{{\left(1-q\right)}^{2}q\gamma_{0}\mu_{0}\psi R{\left(x\right)}^{3}}{\eta^{3}}-\frac{\left(1-q\right)q\gamma_{0}\mu_{0}R{\left(x\right)}^{2}}{\eta^{2}}\right)}{\sum_{i=1}^{\infty }x\exp\left(-rx\right)\exp\left[-\left(\frac{1}{aqR(x)}+\psi V_{0}\left(x\right)\right)\right]\left(1-q\right)\gamma_{0}R\left(x\right)/\eta }$$

where $\eta =\mu_{n}(\gamma_{0}+\mu_{0}\left(1-bqR\left(x\right)\right)$. In this expression, the net reproductive rate is scaled by a non‐linear function associated with the abundance of precursor reproductive cells ($V_{0}$) while the expected age of reproduction is scaled by the precursor reproductive cell maturation ($\gamma_{0}$), functional reproductive cell mortality ($\mu_{n}$), and the abundance of precursor cells ($V_{0}$) (Appendix).

Limiting cases (Appendix) show that the strength of selection for reproductive cell protection is critically dependent on the fundamental levels of resources available and the amount of resource allocated to somatic and reproductive cell maintenance (q).

#### 3.2.2 Reproductive value

Reproductive value (Fisher, 1930; Goodman, 1974, 1982), the relative contribution of individuals (and the associated life history decisions) at different ages to the overall lifetime fitness, can be expressed in terms of the effects of cellular damage on survival and reproduction as:

(14) $$v_{x}=\frac{\exp(r(x-1))}{\exp\left[-\left(\frac{1}{aqR(x)}+\psi V_{0}\left(x\right)\right)\right]}\sum_{t=x}^{\infty }\exp\left(-rt\right)\exp\left[-\left(\frac{1}{aqR(t)}+\psi V_{0}\left(t\right)\right)\right]m\left(t\right).$$

**Figure 3:**
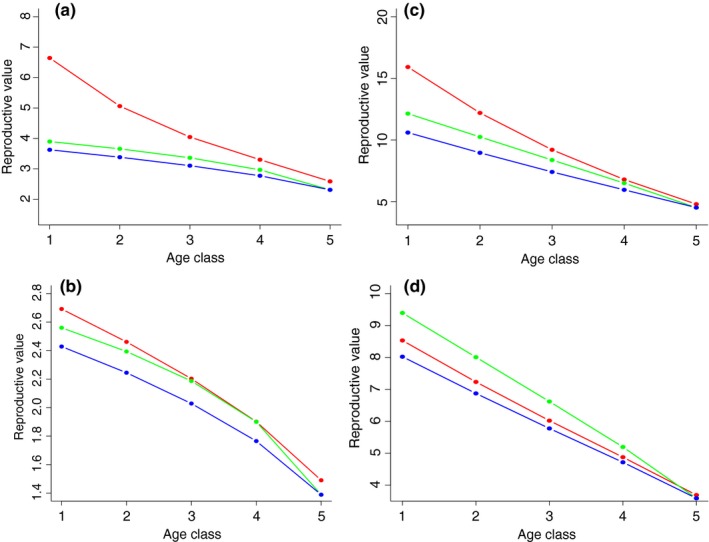
Effects of investment in protecting somatic and/or precursor reproductive cells on reproductive value in (A–B) increasing (r=1.1) or (C–D) decreasing (r=-1.1) populations under different rates of reproductive cell maturation (slow maturation: panels A,C $\gamma_{0}=0.75$; high maturation: panels B,D $\gamma_{0}=2.0$). Lines represent different allocation strategies: (red line: low a=0.1, high b=0.9. green line: high a=0.9, low b=0.1. blue line: low a=0.1, low b=0.1) [Other parameters: q=0.5, $\mu_{0}=0.1$, $\mu_{n}=0.1$, ψ=1.0]

Following the physiological dynamics of reproductive cell production (Equations 1 and 2), different reproductive values are predicted for different combinations of investment in somatic cell protection (a) and precursor reproductive cell protection (b) (Figure 3). As resources decline with age, reproductive value declines with age irrespective of investment patterns. However, these declines in reproductive value can be offset by high investment in protecting somatic and reproductive cells.

Age‐dependent patterns of reproductive value depend on the underlying population (increasing or decreasing) and physiological (high or low reproductive cell development) dynamics. For given physiologies (comparing Figure 3A with 3C and Figure 3B with 3D), declining populations always have higher reproductive value than increasing populations as the relative contribution of individuals to overall fitness is expected to be higher. High contribution of producing offspring early in life is favoured in declining populations.

Under increasing population dynamics (Figure 3A,B), higher reproductive value is expected when relatively more resources are allocated to protect precursor reproductive cells. The value of high investment in protecting precursor reproductive cells is lost as individuals age, particularly when precursor reproductive cell maturation is high (Figure 3B).

Under declining populations, different predictions emerge. When precursor reproductive cell maturation is low (Figure 3C), higher investment in precursor reproductive cells gives rise to higher reproductive value. However, when precursor reproductive cell maturation is high, higher allocation of protection to somatic cells over precursor reproductive cells favors higher reproductive values (Figure 3D). With higher development rates, reproductive cells spend less time in a precursor state hence investments in protecting somatic cells are predicted to yield higher reproductive returns.

### 3.3 Between life history strategies

Predicted fitness (lifetime fitness (r) as a function of resources (R) allocated to cellular maintenance (q)) changes as the allocation to protection of somatic (a) and reproductive cells (b) changes.

Fitness is higher when there is more allocation to protection of somatic cells (a) rather than to protection of reproductive cells (b) as this depends on the survival function (Appendix). Here, survival to age x is the product of $\exp\left(-\frac{1}{aqR(x)}\right)$ and $\exp(-\psi V_{0}\left(x\right))$. The function $\exp\left(-\frac{1}{aqR(x)}\right)$ is the survival costs of protecting somatic cells, and this leads to very low survival as a→0. In contrast, as a→1 (and b→0), survival is maximized (and hence fitness is maximized) as a function of resources allocated to cellular maintenance (q) (Appendix).

**Figure 4:**
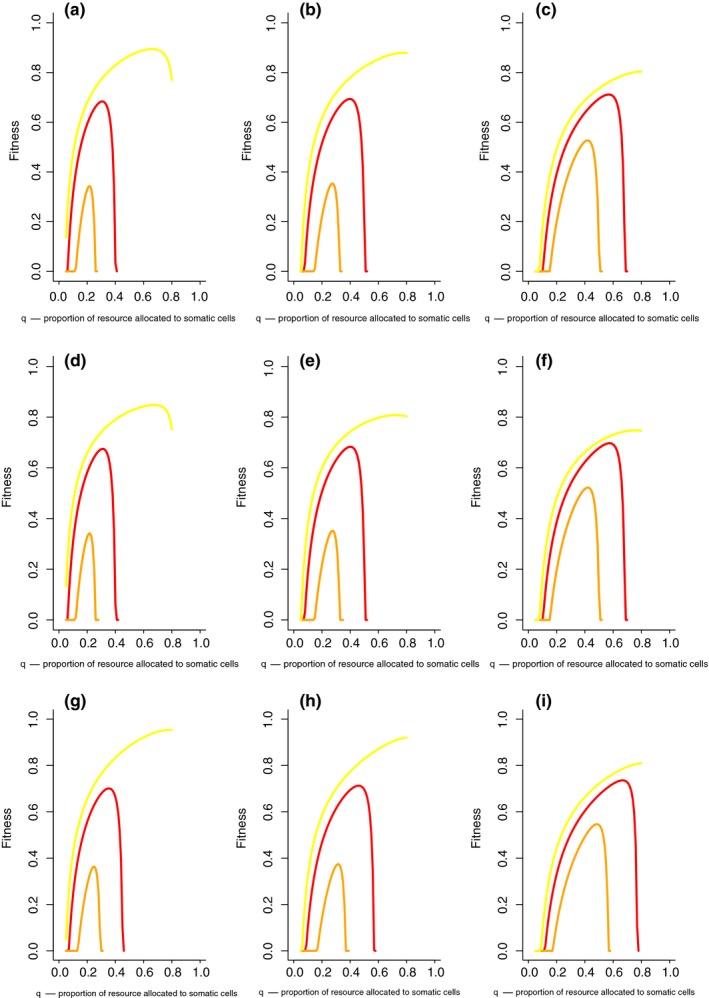
Lifetime fitness as a function of resource allocation (q) to cellular protection and maintenance for different allocations to protecting somatic cells (a) and reproductive cells (b) [a=b (red line); a>b (yellow line); a<b (orange line)] under different resource use functions as the organism ages [(A–C) linear decline in resources, (D–F) accelerating (exponential) decline in resources, (G‐I) decelerating (1‐/exp(‐x)) decline in resources] and physiological demand increase [A,D,G: 1 ‐ (*a* + *b*) = 0; B,E,H: 1 ‐ (*a* + *b*) = 0.2; C,F,I: 1 ‐ (*a* + *b*) = 0.4]. Increasing physiological demand constrains fitness and leads to nonextreme fitness values [Other parameters $\gamma_{0}=0.1$, ψ=0.01, $\mu_{0}=0.1$, $\mu_{n}=0.1$]

Overall, across different life history scenarios of resource uses and physiological demand (1 − (*a* + *b*) > 0), lifetime fitness is always higher when the amount of protection to somatic cells (*a*) is greater than the amount of protection to reproductive cells (*b*) (Figure 4). When the amount of protection to somatic and reproductive cells is equivalent (*a* = *b*), lifetime fitness peaks at intermediate levels of resource allocation to cellular maintenance (*q*). When protection of somatic cells is greater than reproductive cells (*a* > *b*), lifetime fitness, as noted, is higher and peaks at high levels of resource allocation to cellular maintenance (*q*). When protection of somatic cells is less than reproductive cells (*a* < *b*), lifetime fitness, as noted, is lower and peaks at low levels of resource allocation to cellular maintenance (*q*).

Resource use patterns (resources declining linearly with age (Figure 4A–C), accelerating decline with age (Figure 4D–F), decelerating decline with age Figure 4G–I) only affect quantitative patterns in lifetime fitness rather than leading to qualitative differences in allocation decisions and fitness patterns.

In contrast, physiological demand to maintain other cellular functions (1 − (*a* + *b*) > 0) (Figure 4A,D,G: *a* + *b* = 1; Figure 4B,E,H: a+b=0.8; Figure 4C,F,I: a+b=0.6) affects both the quantitative and qualitative patterns in lifetime fitness under resource allocation to cellular maintenance (q). Increasing physiological demand decreases lifetime fitness when somatic cell protection is greater than reproductive cell protection (a>b), increases lifetime fitness when reproductive cell protection is greater than somatic cell protection (a<b) and shifts the peak in lifetime fitness when the amount of protection to somatic and reproductive cells is equivalent (a=b). Increasing physiological demand drives life history strategies toward an equivalent protection allocation strategy (toward a=b) potentially through stabilizing selection on nonextreme values: physiology is constraining fitness.

## 4 DISCUSSION

Here, we have investigated the role of resource allocation and physiological dynamics on life history evolution. We introduce and describe a framework that considers the dynamics of reproductive cell maturation and death, and scale these dynamics up to describe the evolutionary fitness of life history strategies. Our model can provide insights into the evolution of strategies to mitigate cellular damage to different tissue types (somatic, reproductive) depending on how resources are allocated and how resources decline as individuals get older.

Resource acquisition and allocation are central in determining optimal life history strategies. That phenotypes have multiple manifestations through differences in underlying physiologies has been thoroughly appreciated (Tinbergen, 1963). Dynamic resource allocation models (Gadgil & Bossert, 1970; Perrin & Sibly, 1993; Taylor et al., 1974) highlight how general patterns of optimal energy allocation should change with age. However, more recently, McNamara and Houston (2009) argued for more consideration of how physiological mechanisms have evolved and shaped adaptation, rather than considering them purely as constraints. The framework we develop here highlights how physiological dynamics and life history fitness consequences can be explicitly linked to understand adaptations.

Specifically, our focus on cellular protection reveals that the force of selection for protection of precursor reproductive cells is a function of resource input and allocation. Such a link between resource availability and investment in defense has been widely demonstrated. For instance, in a study on *Bicyclus anynana* butterflies, investment in defense increased only under challenging thermal conditions (Beaulieu, Geiger, Reim, Zielke, & Fischer, 2015): Investment in cellular defense reflects prioritization of self‐maintenance over reproduction when the trade‐off is exacerbated by limits on resource acquisition. More recently, work on (rewilded) mice and the interaction between foraging, resource levels, and parasite burdens revealed a complex nexus of interactions mediated by resources, immunity, and ecology (Budischak et al., 2018). Physiological assays of immune function revealed that mice on nutrient poor diets had reduced immune function. However, this did not influence parasite burdens. In field experiments, foraging (on multiple resources) reduced mouse weight loss associated with high parasite burdens (by affecting the physiological controls on feeding). These findings emphasize the importance of measuring physiological (cellular‐ or tissue‐specific) investment patterns to understand the evolutionary outcomes of resource‐mediated trade‐offs.

Our framework highlights how patterns of resource level and allocation affect optimal life history strategies. Here, we have assumed that resources decline as a function of age, but future work should investigate alternative patterns of resource change: for instance, individuals may improve in their ability to acquire resources up until a certain age, or the availability of resources may follow an alternative temporal, seasonal or age‐dependent pattern. This has important implications as life history theory suggests that resource availabilities and allocation decisions affect patterns of senescence (Yearsley et al., 2005), size at maturity (Reznick, Butler, & Rodd, 2001), lifetime fitness (Reznick & Yang, 1993), and investment in levels of immune defenses (Norris & Evans, 2000). All of these have a physiological basis to trade‐offs (Harshman & Zera, 2007; Stearns, 1989; Zera & Harshman, 2001).

Considering the physiological dynamics at the cellular level, we find that investment in protection of precursor reproductive cells increases cellular fitness when mortality rates of these precursor cells is high or when development times are slow. This suggests that species will have higher levels of defenses in their reproductive tissue when cell death rates are high or when there is slow development of these cells. A limitation in testing these ideas is that the specific mechanisms of precursor reproductive cell production may not be known in many systems. Greater empirical attention on understanding reproductive physiologies in a behavioral ecological context is important as this has implications for several predictions about the evolution of defense systems. Our model framework focuses on how organisms avoid damage through allocating resources to repair. Future theoretical work could expand this work to consider how cellular strategies mitigate the effects of damage once it has occurred, such as increased apoptosis of damaged cells (Kirkwood, 2005).

We find that the trade‐off between early and late reproduction, as manifested in investment in reproductive over somatic defenses, depends on the background demographic (population) dynamics. Hoogendyk and Estabrook (1984) highlight that the expected evolutionary effects of earlier reproduction in declining populations are contingent on an appropriate understanding of how development times, mortality rates, and fecundities interact to affect changes in lifetime fitness (r). We show here that as development times and schedules change, this alters the levels of precursor reproductive cell mortality and hence affects both survival and fecundity schedules. Investment in somatic cell maintenance (high a) over reproductive cell protection (low b) leads to higher reproductive values in declining compared to increasing populations. Under varying reproductive cell development, we would predict that with slow development of reproductive cells, investing in protecting reproductive cells would maximize reproductive value, and hence lifetime fitness. Conversely, fast development favors investing less in protecting precursor reproductive cells and, instead, maintaining high somatic cell survival. However, it is likely that these effects are not independent of other life history processes. For instance, Buttermer, Abele, and Costantini (2010) have suggested that high rates of cell renewal, observed in some short‐lived marine invertebrates, reduce the need for protection of somatic cells. Unraveling this sort of biological detail on the role of investment in defense and reproductive physiology might be best approached through comparative analyses of different species (Rey, Pélisson, Bel‐Venner, Voituron, & Venner, 2015; Strahl & Abele, 2010).

We consider fitness at multiple levels—cellular, individual, population—and find complex patterns for resource allocation strategies at each level of organization. Our results highlight the utility of such an approach for measuring fitness, as any single individual component of fitness may not provide the complete overview of life history evolution. In considering the Euler–Lotka measure of lifetime (population‐level) fitness, we take into account the effects of investment in cellular protection on both the survival and reproductive contributions to fitness. This allows us to consider complex relationships between resource input, allocation and mortality or fecundity: for example, survival has a hump‐shaped relationship for a fixed resource allocation strategy as it is a function of both somatic damage (which decreases with increasing resources) and costs of reproduction (which increase with increasing resources, as more reproductive cells are generated). While empirical studies do not always calculate age‐specific schedules of both survival and reproduction, we believe that these data would provide an appropriate population‐level measure of fitness to allow the development of a comprehensive understanding of the life history consequences of resource allocation and physiological trade‐offs.

In summary, we present a theoretical framework to investigate the evolutionary consequences of investment in cellular defense, by linking the dynamics of reproductive cell production and death to population‐level fitness, which incorporates age‐specific schedules of survival and fecundity. Our approach raises a number of insights about how patterns of resource acquisition, allocation and mitigation of damage in somatic or reproductive tissue can generate contrasting life history strategies.

This framework emphasizes possibilities for future empirical and theoretical work. Future empirical studies should consider the physiological mechanisms underlying resource allocation at a cellular level, as well as measuring fitness in a way that takes account of survival and fecundity across the whole life history of an organism. Future theoretical models can build on our framework to consider trade‐offs amongst life history parameters, including growth as well as reproduction and repair, and other schedules of how resource input changes with age. More generally, the framework we develop provides a novel way in which to link physiological mechanism and dynamics to components of life history, fitness, and adaptation.

## CONFLICT OF INTERESTS

We declare we have no conflict of interests.

## AUTHOR CONTRIBUTIONS

Both authors conceived the study. Model development and analysis was undertaken by MBB and both authors contributed to writing the manuscript.

## Data Availability

The scripts and data supporting the results presented in this the paper are archived with the Open Science Framework at osf.io/ac79p/.

## APPENDIX 1

### STABILITY OF REPRODUCTIVE ALLOCATION MODEL

The stability of the equilibrium state (Equations 3 and 4) can be found by taking a Taylor expansion of Equations 1 and 2 around this equilibrium state and evaluating the roots (ω) of the resulting characteristic equation. The Jacobian matrix for the reproductive cell dynamics is as follows:

$$\left(\begin{array}{cc} -\omega -\gamma_{0}-\mu_{0}\left(1-bqR\right) & 0\\ \gamma_{0} & -\omega -\mu_{n} \end{array}\right).$$

Taking the determinant of this matrix gives the following characteristic equation:

$$\omega^{2}+\omega (\mu_{n}+\gamma_{0}+\mu_{0}\left(1-bqR\right)+\mu_{n}\left(\mu_{0}\left(1-bqR\right)+\gamma_{0}\right)=0.$$

Routh–Hurwitz stability criteria for a 2D system (where all coefficients in the quadratic expression have to be greater than 0 to ensure that small perturbation decay away) imply this system is stable when:

$$\mu_{n}+\gamma_{0}+\mu_{0}\left(1-bqR\right)>0$$

and

$$\mu_{n}(\mu_{0}\left(1-bqR\right)+\gamma_{0}>0.$$

### DERIVATION OF THE EXPRESSION FOR CELLULAR FITNESS

An expression for cellular fitness (Equation 11) can be derived from linear stability theory. By linearizing the reproductive cell dynamics (Equations 1 and 2) around zero and determining the conditions which give rise to positive growth, the dominant eigenvalue provides a measure of this (cellular) component of fitness.

For the reproductive cell dynamics, the linearized dynamics (in matrix form) are:

$$\left(\begin{array}{cc} -\lambda -\gamma_{0}-\mu_{0}\left(1-bqR\right) & 0\\ \gamma_{0} & -\lambda -\mu_{n} \end{array}\right)$$

where λ is the measure of cellular fitness (Table 1). Taking the determinant of this Jacobian matrix, the resulting characteristic equation is as follows:

$$\lambda^{2}+\lambda \left(\gamma_{0}+\mu_{0}+\mu_{n}-bqR\mu \right)+\gamma_{0}\mu_{n}-bqR\mu_{0}\mu_{n}.$$

Setting this expression to zero and solving for (the leading eigenvalue), λ, yields:

$$\lambda =\left(bqR-1\right)\mu_{0}-\gamma_{0}$$

as the (cellular) component of fitness. This is a measure for positive population net growth when the strategy is rare.

### DERIVATION OF FORCE OF SELECTION ON LEVELS OF PROTECTION FOR A SINGLE AGE CLASS

#### Force of selection on *a*

Here we outline the derivation of the force of selection associated with changes in the levels of protection for somatic and reproductive cells for a single age class. The Euler–Lotka expression for a single age class is

$$\exp\left(-rx\right)\exp\left[-\left(\frac{1}{aqR(x)}+\psi V_{0}\left(x\right)\right)\right]V_{n}\left(x\right)=1.$$

where $l\left(x\right)=\exp\left(-\left(\frac{1}{aqR(x)}+\psi V_{0}\left(x\right)\right)\right)$ and $m\left(x\right)=V_{n}\left(x\right)$. To derive the force of selection on levels of protection on somatic cells, we take the derivative of the Euler–Lotka equation with respect to a. That is

$$\frac{\partial \exp\left(-rx\right)\exp\left(-\left(\frac{1}{aqR(x)}+\psi V_{0}\left(x\right)\right)\right)V_{n}\left(x\right)}{\partial a}=0.$$

Using the chain rule this can be expanded to:

$$\begin{array}{cc} & \left[\exp\left(-rx\right)\frac{\partial \exp\left(-\left(\frac{1}{aqR(x)}+\psi V_{0}\left(x\right)\right)\right)}{\partial a}V_{n}\left(x\right)\right]\\ & \;+\left[\exp\left(-\left(\frac{1}{aqR(x)}+\psi V_{0}\right)\right)\frac{\partial \exp(-rx)}{\partial r}\frac{\partial r}{\partial a}V_{n}\left(x\right)\right]=0 \end{array}$$

and worked through to give:

$$\begin{array}{cc} & \left[\exp\left(-rx\right)\frac{\exp\left(-\left(\frac{1}{aqR(x)}+\psi V_{0}\left(x\right)\right)\right)\left(1-q\right)\gamma_{0}}{a^{2}q\mu_{n}\left(\gamma_{0}+\mu_{0}\left(1-bqR\left(x\right)\right)\right)}\right]\\ & \;-\left[\exp\left(-\left(\frac{1}{aqR(x)}+\psi V_{0}\left(x\right)\right)\right)x\exp\left(-rx\right)\frac{\partial r}{\partial a}V_{n}\left(x\right)\right]=0 \end{array}$$

So,

$$\frac{\partial r}{\partial a}=\frac{\exp\left(-rx\right)\frac{\exp\left(-\left(\frac{1}{aqR(x)}+\psi V_{0}\left(x\right)\right)\right)\left(1-q\right)\gamma_{0}}{a^{2}q\mu_{n}\left(\gamma_{0}+\mu_{0}\left(1-bqR\left(x\right)\right)\right)}}{x\exp\left(-rx\right)\exp(-\left(\frac{1}{aqR(x)}+\psi V_{0}\left(x\right))\right)V_{n}\left(x\right)}$$

This can be simplified to:

$$\frac{\partial r}{\partial a}=\frac{1}{a^{2}qxR\left(x\right)}$$

as the expected number of precursor reproductive cells is independent of the level of protection on somatic cells (a).

#### FORCE OF SELECTION ON *b*

To derive the force of selection on levels of protection on reproductive cells, we take the derivative of the Euler–Lotka equation with respect to *b*.

$$\frac{\partial \exp\left(-rx\right)\exp\left(-\left(\frac{1}{aqR(x)}+\psi V_{0}\left(x\right)\right)\right)V_{n}\left(x\right)}{\partial b}=0.$$

Working through the steps of implicit differentiation of this equation yields:

$$\frac{\partial r}{\partial b}=-\frac{q\mu_{0}R\left(x\right)\left(\left(bq\mu_{0}+\psi -q\psi \right)R\left(x\right)-\gamma_{0}-\mu_{0}\right)}{x{\left(\gamma_{0}+\mu_{0}-bq\mu_{0}R\left(x\right)\right)}^{2}}.$$

### DERIVATION OF FORCE OF SELECTION ON PROTECTION *a* and *b* for across all age classes

Here we outline the derivation of the force of selection associated with changes in the levels of protection for somatic and reproductive cells across all age class. The discrete version of Euler–Lotka expression is given by:

$$\sum_{x=1}^{\infty }\exp\left(-rx\right)\exp\left[-\left(\frac{1}{aqR(x)}+\psi V_{0}\left(x\right)\right)\right]V_{n}\left(x\right)=1.$$

where $l\left(x\right)=\exp\left(-\left(\frac{1}{aqR(x)}+\psi V_{0}\left(x\right)\right)\right)$ and $m\left(x\right)=V_{n}\left(x\right)$.

#### FORCE OF SELECTION ON *a*

To derive the force of selection on levels of protection on somatic cells, we take the derivative of the Euler–Lotka equation with respect to a using methods of implicit differentiation. That is

$$\frac{\partial \sum_{x=1}^{\infty }\exp\left(-rx\right)\exp\left(-\left(\frac{1}{aqR(x)}+\psi V_{0}\left(x\right)\right)\right)V_{n}\left(x\right)}{\partial a}=0.$$

Using the chain rule this can be expanded to:

$$\begin{array}{cc} & \sum_{x=1}^{\infty }\left[\exp\left(-rx\right)\frac{\partial \exp\left[-\left(\frac{1}{aqR(x)}+\psi V_{0}\left(x\right)\right)\right]V_{n}\left(x\right)}{\partial a}\right]\\ & \;+\sum_{x=1}^{\infty }\left[\exp\left[-\left(\frac{1}{aqR(x)}+\psi V_{0}\left(x\right)\right)\right]V_{n}\left(x\right)\frac{\partial \exp(-rx)}{\partial r}\frac{\partial r}{\partial a}\right]=0 \end{array}$$

Working this through with appropriate substitutions for $V_{0}\left(x\right)$ and $V_{n}\left(x\right)$ yields:

$$\frac{\partial r}{\partial a}=\frac{\sum_{i=1}^{\infty }\exp\left(-rx\right)\exp\left[-\left(\frac{1}{aqR(x)}+\psi V_{0}\left(x\right)\right)\right]V_{n}\left(x\right)}{a^{2}qR\left(x\right)\sum_{i=1}^{\infty }x\exp\left(-rx\right)\exp\left[-\left(\frac{1}{aqR(x)}+\psi V_{0}\left(x\right)\right)\right]V_{n}\left(x\right)}.$$

#### FORCE OF SELECTION ON *b*

To derive the force of selection on levels of protection on reproductive cells, we take the derivative of the Euler–Lotka equation with respect to b and used the rules of implicit differentiation to solve the resulting equation for $\frac{\partial r}{\partial b}$.

Working through the steps of implicit differentiation of this equation yields:

$$\frac{\partial r}{\partial b}=-\frac{\sum_{i=1}^{\infty }\exp\left(-rx\right)\exp\left[-\left(\frac{1}{aqR(x)}+\psi V_{0}\left(x\right)\right)\right]\left(\frac{{\left(1-q\right)}^{2}q\gamma_{0}\mu_{0}\psi R{\left(x\right)}^{3}}{\eta^{3}}-\frac{\left(1-q\right)q\gamma_{0}\mu_{0}R{\left(x\right)}^{2}}{\eta^{2}}\right)}{\sum_{i=1}^{\infty }x\exp\left(-rx\right)\exp\left[-\left(\frac{1}{aqR(x)}+\psi V_{0}\left(x\right)\right)\right]\left(1-q\right)\gamma_{0}R\left(x\right)/\eta }$$

where $\eta =\mu_{n}(\gamma_{0}+\mu_{0}\left(1-bqR\left(x\right)\right)$.

### CHANGES IN THE SURVIVAL L(X) SCHEDULE ON FITNESS

The survival schedule (l(x)) is a product of how resources are allocated to maintenance of somatic cells and the availability of resources to reproductive cells. We assume that resource allocation to somatic cells declines and is described by the function:

$$\exp\left(-\frac{1}{aqR(x)}\right).$$

Similarly, the availability of resources affects the dynamics of the reproductive cells and we assume that as the abundance of precursor cell increases through high levels of resource allocation, this has a negative consequence on survival. This biology is described by the function:

$$\exp\left(-\psi V_{0}\right)$$

where ψ is a scaling linking the number of precursor reproductive cells to costs on survival and $V_{0}$ is the expected abundance of precursor reproductive cells. The overall survival schedule is the product of these two functions:

$$l\left(x\right)=\exp\left[-\frac{1}{aqR(x)}-\psi V_{0}\right].$$

Under a limiting case, when a→0 and b→1 then:

$$l\left(x\right)\to \exp\left[-\infty -\frac{(1-q)R(x)\psi }{\gamma_{0}+\left(1-bqR\left(x\right)\right)\mu_{0}}\right]$$

So l(x)→0 in this limit. Low investment in protecting somatic cells limits survival. In contrast when *a* = 1 and *b* = 0 then:

$$l\left(x\right)=\exp\left[-\frac{1}{qR(x)}-\frac{(1-q)R(x)\psi }{\gamma_{0}+\mu_{0}}\right]$$

In this limit, high investment in protecting somatic cells ensure overall survival. Furthermore, under this limit (*a* = 1, *b* = 1) the derivative of this survival function with respect to resource allocation (*q*) is as follows:

$$\frac{\partial (l(x))}{\partial q}=\exp\left[-\frac{1}{qR(x)}-\frac{(1-q)R(x)\psi }{\gamma_{0}+\mu_{0}}\right]\left[\frac{1}{q^{2}R\left(x\right)}+\frac{R(x)\psi }{\gamma_{0}+\mu_{0}}\right]$$

without losing generality, if $\gamma_{0}=1$,ψ=1.0 and $\mu_{0}=0$, then survival is maximized (and hence fitness is maximized) when:

$$q=\frac{-R\left(x\right)+R{\left(x\right)}^{2}}{2R{\left(x\right)}^{2}}+R\left(x\right)\sqrt{5-2R\left(x\right)+R{\left(x\right)}^{2}}.$$

There is an optimal value of *q* for high protection of somatic cells compared to reproductive cells (*a* ≫ *b*) which is dependent on the underlying level of resource (*R*) at age *x*. In contrast, *a* ≪ *b*, fitness is compromised by very low survival.
